# Ethical and legal constraints to children’s participation in research in Zimbabwe: experiences from the multicenter pediatric HIV ARROW trial

**DOI:** 10.1186/1472-6939-13-17

**Published:** 2012-07-20

**Authors:** Mutsa Bwakura-Dangarembizi, Rosemary Musesengwa, Kusum J Nathoo, Patrick Takaidza, Tawanda Mhute, Tichaona Vhembo

**Affiliations:** 1 Department of Pediatrics and Child Health, University of Zimbabwe College of Health Sciences, Box A178, Avondale, Harare, Zimbabwe; 2 Medical Research Council of Zimbabwe, P.O. Box CY489, Causeway, Harare, Zimbabwe; 3 University of Zimbabwe Clinical Research Centre, P.O. Box A1578, Avondale, Harare, Zimbabwe

## Abstract

**Background:**

Clinical trials involving children previously considered unethical are now considered essential because of the inherent physiological differences between children and adults. An integral part of research ethics is the informed consent, which for children is obtained by proxy from a consenting parent or guardian. The informed consent process is governed by international ethical codes that are interpreted in accordance with local laws and procedures raising the importance of contextualizing their implementation.

**Findings:**

In Zimbabwe the parental informed consent document for children participating in clinical research is modeled along western laws of ethics and requires that the parent or legally authorized representative provide consent on behalf of a minor. This article highlights the experiences and lessons learnt by Zimbabwean researchers in obtaining informed consent from guardians of orphaned children participating in a collaborative HIV clinical trial involving the Medical Research Council, United Kingdom and four centers, three of which are in Uganda. Researchers were faced with a situation where caregivers of orphaned children were not permitted to provide informed consent for trial participation. The situation contrasted with general clinical practice where consent for procedures on orphans is obtained from their caregivers who are not legal guardians.

**Conclusion:**

The challenges faced in obtaining informed consent for orphans in this clinical trial underscores the need for the Zimbabwe ethics committee to develop an ethical and legal framework for pediatric research that is based on international guidelines while taking into account the cultural context. The Medical Research Council of Zimbabwe has since started the process that is expected to involve critical stakeholders namely the community including children, ethicists, the legal fraternity and researchers.

## Background

Research in human participants is governed by international ethical codes and legislation derived from the basic moral principles of autonomy, beneficence and justice. The responsibility for research participant protection lays with ethics committees (EC) and institutional review boards (IRB) whose mandate is to review research projects for their scientific merit and clinical usefulness. One of the key responsibilities of ECs in protecting human participants is review of the informed consent process to ensure that accurate information about the study and its purpose are conveyed, including known risks, benefits and alternatives; that procedures are disclosed, and questions are answered, thus enabling the participant to make an informed decision about whether or not to participate [1]. Certain groups are considered vulnerable research participants because they may have insufficient power, intelligence, education, resources, strength or other necessary attributes to protect their own interests [1]. Children, defined as persons who have not attained the legal age of consent, belong to this vulnerable group and consent is obtained by proxy from a parent or legally appointed guardian in accordance with local laws or established procedures [1] The parents or legal guardian are expected to act in the best interests of the child throughout the research. An additional challenge faced in conducting research that involves children is achieving the balance between the social good that comes from children’s participation and offering the appropriate level of protection to them. Major research groups and professional bodies worldwide such as the National Institutes of Health (NIH) in the USA, The Medical Research Council (MRC) in the UK and The Royal College of Pediatrics and Child Health (RCPCH) in the UK, have published policy statements on the importance of assessing health-care interventions for use in children through randomized controlled trials [2]. This has arisen from the understanding that children are physiologically, developmentally and emotionally different from adults and developing treatments for children by extrapolating results from adult studies may actually impose harm [3]. Protection of children from exploitation has remained central leading to the inclusion of assessing risk versus benefit of research involving children. The US Federal Policy for the Protection of Human Subjects has defined four categories where research in children may be conducted [4]. These are minimal risk, greater than minimal risk and prospect of direct benefit, greater than minimal risk and no prospect of direct benefit and research not otherwise approvable. Ethics committees are given the responsibility for determining these risk categories. The MRC Ethics Guide for medical research involving children also upholds minimizing risk and assessing potential benefits of research not only for the participant but for children as a whole [5] Such frameworks may not be developed to the same level and tend to lag behind in developing countries where an increasing amount of pediatric research takes place. An example is the separation of ‘therapeutic’ and non-therapeutic research’ which is no longer used in the Declaration of Helsinki (2002) but is still in use in some countries including Zimbabwe. The recent globalization of health has seen more research involving children taking place in developing countries through collaborations between developed and developing countries to the mutual benefit of both. This move is an attempt to redress the global inequity in research dubbed the ‘10/90’ gap in which less than 10% of the estimated US$70 billion spent annually on health research addresses the conditions that account for 90% of the global disease burden, measured by the number of disability-adjusted-life-years [6]. Generally, collaborative studies must have ethical approval from the sponsoring country as well as the country or institutions where the research will be conducted. This brings together ethical standards from different socio-cultural backgrounds. Ethics committees in both the sponsor country and the host country have the responsibility to ensure scientific and ethical review as well as the authority to deny approval of research protocols that fail to meet their scientific or ethical standards [2]. Ethics committees in the host country must, in addition, determine whether the research objectives are responsive to the local health needs and priorities. According to the Council for International Organizations of Medical Sciences (CIOMS) guidelines of 2002, the EC in the host country can be expected to have greater competence for reviewing the detailed plans for compliance, in view of its better understanding of the cultural and moral values of the population in which it is proposed to conduct the research [2]. The host country is also likely to be in a better position to monitor compliance during the course of a study. The importance of taking into account the context within which research is conducted has been highlighted in research conducted in other developing countries. Individual decision-making characterizes the informed consent process in developed western countries unlike the family and community participation in some Asian and African countries [7,8]. South Africa is one developing country that has made some progress in addressing consenting issues for children in their Children’s Act of 2005 [9] that allows children to consent independently to health-related interventions such as male circumcision and HIV testing. In addition to parents and legal guardians, the act has made provision for caregivers such as grandparents or other relatives who do not have parental responsibilities and rights but are able to provide proxy consent on behalf of minors [10]. This is an innovative development that acknowledges the reality that a significant number of children will be taken care of by caregivers such as grandparents or other relatives. The legal framework for health research in South Africa does not however have provision for children to consent to any form of health research and limits proxy consent to parents and legal guardians leaving research ethics committees to rely on the principles of ethical guidelines.

In this paper, we discuss the experiences faced by researchers in obtaining informed consent for orphaned children in Zimbabwe and how this highlighted the need to develop an ethico-legal framework to guide research-involving minors that is sensitive to the socio-cultural environment. We also offer some recommendations of issues to be taken into consideration when ethics committees develop these guidelines.

## Discussion

### The ARROW clinical trial

Antiretroviral Research for Watoto (ARROW) is a phase III multicenter clinical trial evaluating monitoring and first-line antiretroviral therapy strategies in 1200 HIV-1 infected children in Uganda and Zimbabwe [11]. This study is co-sponsored by the United Kingdom (UK) Medical Research Council (MRC) and the UK Department for International Development (DFID). A Trial Steering Committee (TSC) comprising independent members from the three countries, the principal investigators and an independent chair govern the ARROW trial. The TSC supervises the progress of the trial towards its interim and overall objectives, focusing on adherence to the protocol, Good Clinical Practice, patient safety and the consideration of new information [11]. The Researchers from United Kingdom, Uganda and Zimbabwe were involved in drafting the main protocol and developing information sheets together with consent forms for parents or caregivers as well as children who had reached the level of assent. These documents were translated into the respective local languages before submission to Ethics Committees for review and approval. ARROW received approval from the Ugandan EC and Zimbabwean ECs in February and March 2006 respectively.

### Current governance of research and informed consent for children in Zimbabwe

The Medical Research Council of Zimbabwe (MRCZ), the National Ethics Committee was established in 1974 under the Research Act of 1959 and Government Notice Number 225 of 1974 in order to provide health researchers and institutions conducting health research with independent ethical advice on research [12]. It is composed of scientists, medical experts, ethicists, lawyer and religious and community representatives, making a total of 14 members. The Medicines Control Authority of Zimbabwe (MCAZ) regulates all clinical trials that are conducted in Zimbabwe in terms of Part III of the Medicines and Allied Substances Control Act [Chapter 15:03] [13]. There are no specific laws that are dedicated to research involving children in Zimbabwe. However there are two acts that relate to consent of minors in clinical trials and in organ donation or removal. The Medicines and Allied Substances Control Act Chapter 3:20 pertains to consent of minors in clinical trials and says;

“Where the Council grants written authorisation under section eighteen for the conduct of a trial of a drug, no such trial shall take place until -

b) in the case of a medicine for the treatment of minors or persons under legal disability, the voluntary written consents of their parents or legal guardians, as the case may be, have been freely obtained by the person conducting the trial.

The Anatomical Donations and Post-Mortem Examinations Act [14] says;

“Replacement tissue may be removed from the body of a living person for scientific purposes or therapeutic purposes-"

12 b) in the case of a person under the age of eighteen years, whether or not he is capable of expressing his consent, with the written consent of his parents or guardian:

Both these acts are not specific for research involving children and do not refer to the child’s assent or the child’s best interest. In addition the term legal guardian is interpreted using legal terminology.

The MRCZ requires that parental informed consent be obtained from the parent of the child or a legally authorized representative. A third party can be appointed guardian of a minor child where there is no natural parent alive or where the natural parent may be alive but it is not in the best interests of the child that they be vested with guardianship of the child and where the available natural guardian does not have the capacity to act as such. The appointment of such a third party is made through a court application outlined below.

• A relative or person having the care and custody of the child lodges an application with the clerk of court for the appointment of a person as guardian of the minor with the following accompanying documents:

• Death certificates to prove that the child’s parents were both deceased.

• The child’s birth certificate.

• Four supporting affidavits, two each from the maternal and paternal relatives

The clerk of court would then publish in the Government Gazette and any other national newspaper circulating in the area where the minor resides calling upon any person interested in the matter to appear before the children’s court on a date specified in the notice and specifying the name of a person proposed for appointment as the guardian. The time period given for the response is not less than 7 days and not more than 30 days after publication of the notice. The clerk of court then issues a letter of appointment to the person appointed as guardian specifying the powers, rights and privileges conferred upon that person. In situations where the consenting parent dies, the statutes regulating the appointment of guardians come into effect. These are the Guardianship of Minors Act, Child Protection and Adoption Act and Administration of Deceased Estates [15,16]. In cases where there is a surviving parent, she/he takes over guardianship and provides the consent. If the consenting parent who was a sole guardian dies, he/she is expected to have had a will with provision for a guardian. Writing of wills is also not a common practice in Zimbabwean African culture. Where the child is left in the custody of relatives who are taking care of the child, the responsible relative for all practical purposes assumes the guardianship of the child, but must be confirmed by the court in terms of section 9 of the Children’s Act. In Zimbabwean culture, the legal route is rarely pursued where custody of children is concerned. A child is placed in the custody of a relative or relatives upon the death of the parents following consultation with the extended family. This guardian takes over parental responsibility of the child including schooling, health care and in the case of the ARROW trial, also took on consent for participation in research.

### Challenges experienced in the ARROW trial

Screening and enrolment into the ARROW clinical trial started rapidly in Uganda; however in Zimbabwe, it became apparent that a substantial number of potential research participants were orphans (120/400; 30%) whose relatives wanted them to be involved in the study but could not because of the requirement by the Law (Guardianship of Minors Act Chapter 5:08) of Zimbabwe for consent from a parent or legal guardian. The researchers learnt that Zimbabwean law only recognized a legal guardian as one who had been given custody of the child by a court. Further enquiry from the legal fraternity and children’s rights groups revealed that the process was both lengthy and costly (estimated cost of USD100 excluding legal fees). This legal process was communicated to the caregivers of potential ARROW participants most of whom were not only unaware of the existence of legal guardianship requirements but were also unwilling to engage in the process. The researchers quickly realized that the unwillingness to apply for legal guardianship was not related to the documentation required e.g. birth certificates as these could be obtained easily through the government system. Death certificates are also a legal requirement in Zimbabwe. Only 1/120 (0.08%) orphans had a guardian appointed by the court. In traditional Zimbabwean African culture, children are viewed as belonging to the community and surviving relatives are expected to take care of a deceased relative’s children. This process does not require formalization through the courts. An adult relative is usually identified as the custodian following the parents’ death with the realization that caring for these children is a shared responsibility. Though Zimbabwe is a patrilineal society, most of the orphaned children were in the custody of their maternal relatives. This may have been a reflection of the young age of the children and the need for maternal input from the mother’s side of the family. The researchers who were all Zimbabweans were constrained by the realization that while caregivers of children could provide consent for treatment including surgery in general medical practice, this was not so in research. They were now faced with a situation where a substantial number of children whose relatives were willing and enthusiastic to provide informed consent for trial participation would be excluded from the clinical trial. This was at a time when the national ART roll-out for children had only just started and was characterized by long waiting periods in a health care delivery system that was going through major challenges. The EC was informed of the challenges that were being faced and how the legal requirement could potentially disadvantage this group of children who were already vulnerable by virtue of being orphans. Among their considerations was the acknowledgement that there was no framework governing research involving children, the importance of not stigmatizing orphans by denying them participation in research that could potentially benefit them and the absence of direct benefits accruing to the caregivers. They also considered what would be in the best interests of the child as compared to fulfilling the requirements of the law. While underscoring the importance of protecting children in research, the EC eventually made a decision to provide a ‘waiver’ of the legal guardianship requirement, but requested that care-givers sign an affidavit stating that they understood the high level of commitment required of them during the ARROW clinical trial. Custodians who were recognized by the family provided consent for the children’s participation in the study. The site proceeded to implement the recommendation and wherever possible engaged both the maternal and paternal sides of an orphaned child’s families in the information giving and consenting process. The site was able to enroll 400 children, 40% were orphans into the trial over the planned 15-month period.

During the course of the study, participants who lost a surviving parent or guardian had to be re-consented for continued participation by another custodian appointed by the family. This re-consenting process would once again include a child’s paternal and maternal relatives as far as possible. Involvement of both sides of a child’s family proved to be an asset because it ensured continuity of care whenever the child visited other relatives (a frequent occurrence) or there was change in guardianship during the course of the trial. A total of 6 children lost a surviving parent during the course of the clinical trial and were consented for continuation in the trial by relatives agreed upon by the family.

### Recommendations

1. While it is generally accepted that informed consent for children’s participation in research is obtained through a proxy namely a parent or legal guardian, the Zimbabwe ethics committee had not foreseen the challenge that researchers faced in adhering to ethical principles within a cultural context that did not recognize or practice legal adoption of orphaned children. An ethical approach that is culturally sensitive and yet adheres to universal ethical standards of child health research [7] should be adopted in the development of an ethico-legal framework that guides research-involving children. Researchers should also familiarize themselves with the requirements for informed consent for vulnerable groups that include children. Dialogue has begun between the Zimbabwe ethics committees, researchers and the legal fraternity with a view to developing this framework.

2. The ARROW clinical trial demonstrated cultural sensitivity in obtaining informed consent for orphaned children by engaging the extended family. Shaibu [7] also suggests this family-centered approach to decision making from her experience in obtaining informed consent in Botswana. It is therefore recommended that the Zimbabwe ethics committee find ways of obtaining community input in the process of drafting the framework that will govern pediatric research. In this age of international collaborative research, it is important for sponsors to have an appreciation of the cultural contexts within which the studies will be conducted. The consideration of ‘parental responsibilities and rights’ of caregivers by the South Africans in their revised Children’s Act could also inform the debate on consenting issues for research involving children who do not have biological parents.

### Summary

The globalization of research particularly in HIV/AIDS calls for an urgent need for developing countries such as Zimbabwe to develop a framework for pediatric research that is based on international ethical guidelines but takes into account the context of local cultural, medical and legal systems. The experience in ARROW, one of the large HIV pediatric clinical trials in Africa, has revealed some deficiencies in the ethical and legal framework governing research-involving children. Subsequent to ARROW, a number of research teams have made similar representations regarding obtaining informed consent for orphans in clinical trials prompting the Medical Research Council of Zimbabwe to re-examine the laws and policies governing research involving children in Zimbabwe with the assistance from the Attorney General’s office.

## Abbreviations

IRB: Institutional Review Board; MRCZ: Medical Research Council of Zimbabwe; HIV: Human Immune deficiency Virus; ARROW: AntiRetroviral Research fOr Watoto; CIOMS: Council for International Organizations of Medical Sciences; WHO: World Health Organization; EC: Ethics Committee.

## Competing interests

The authors declare that they have no competing interests.

## Authors’ contributions

MBD prepared the manuscript with contributions from RM, KN, TM, TV and PT, KN and MBD are the Principal Investigator and Co-principal investigators for the ARROW clinical trial in Zimbabwe. RM is the National Coordinator of the Medical Research Council of Zimbabwe. PT is a practicing lawyer and member of the Medical Research Council of Zimbabwe. TM and TV are medical officers on the ARROW trial who were directly involved in obtaining informed consent for the trial participants. All authors read and approved the final manuscript.

## Pre-publication history

The pre-publication history for this paper can be accessed here:

http://www.biomedcentral.com/1472-6939/13/17/prepub

## References

[B1] CIOMS International Ethical Guidelines for Biomedical Research Involving Human Subjects. Geneva 2002 14983848

[B2] Diekema DS Conducting ethical research in Pediatrics: A brief historical overview and review of Pediatric regulations J Pediatr 2006 149 S3 S11 10.1016/j.jpeds.2006.04.043 16829239

[B3] Caldwell PH Murphy SB Butow PN Craig JC Clinical Trials in Children Lancet 2004 364 803 811 10.1016/S0140-6736(04)16942-0 15337409

[B4] The Code of Federal Regulations and ICH Guidelines 2006

[B5] MRC Ethics Guide Medical Research Involving Children 2004

[B6] 10/90 Report on Health Research 2000 2000 Geneva: Global Forum for Health Research http://www.globalforumhealth.org

[B7] Shaibu S Ethical and Cultural Considerations in Informed Consents in Botswana Nurs Ethics 2007 14 503 509 10.1177/0969733007077884 17562729

[B8] Mystakidou K Panagioutu I Kastaragakis S Tsilika E Parpa E Ethical and practical challenges in implementing informed consent in HIV/AIDS clinical trials in developing or resource-limited countries Journal of Social Aspects of HIV/AIDS 2009 6 46 57 10.1080/17290376.2009.9724930 19936406

[B9] Government Gazette Republic of South Africa 2006 492 28944

[B10] Strode AE Slack CM Using the concept of ‘parental responsibilities and rights’ to identify adults able to provide proxy consent to child research in South Africa South African Journal of Bioethics and Law 2011 4 69 73

[B11] Antiretroviral Research fOr Watoto Governancehttp://www.arrowtrial.org/g_the_governing_committees.asp [accessed 24 March 2011]

[B12] http://www.mrcz.org.zw [accessed 24 March 2011]

[B13] Government of Zimbabwe The Medicines and Allied Substances Control Act. Chapter 15:03. Section 3:20 (b) 2000

[B14] Government of Zimbabwe The Anatomical Donations and Post-Mortem Examinations Act. No 34, Section 12 (b) 1976

[B15] Government of Zimbabwe Children’s Protection and Adoption Act. Chapter 5:06

[B16] Government of Zimbabwe Guardianship of Minors Act. Chapter 5:08 1996

